# Risk factors for development of pulmonary arterial hypertension in Australian systemic sclerosis patients: results from a large multicenter cohort study

**DOI:** 10.1186/s12890-016-0296-z

**Published:** 2016-09-27

**Authors:** Kathleen Morrisroe, Molla Huq, Wendy Stevens, Candice Rabusa, Susanna M. Proudman, Mandana Nikpour, the Australian Scleroderma Interest Group (ASIG), C. Hill, S. Lester, G. Ngian, M. Nikpour, S. Proudman, M. Rischmueller, J. Roddy, J. Sahhar, W. Stevens, G. Strickland, J. Walker, J. Zochling

**Affiliations:** 1 Department of Medicine, The University of Melbourne at St Vincent’s Hospital (Melbourne), 41 Victoria Parade, Fitzroy, 3065 VIC Australia; 2 Department of Rheumatology St Vincent’s Hospital (Melbourne), 41 Victoria Parade, Fitzroy, 3065 VIC Australia; 3 Department of Rheumatology, Royal Adelaide Hospital, North Terrace, Adelaide, SA 5000 Australia; 4 Discipline of Medicine, University of Adelaide, Adelaide, SA 5000 Australia

**Keywords:** Systemic sclerosis, Pulmonary arterial hypertension, Incidence, Prevalence, Risk factors

## Abstract

**Background:**

Pulmonary arterial hypertension (PAH) is the leading cause of mortality in patients with systemic sclerosis (SSc). We sought to determine the incidence, prevalence and risk factors for PAH development in a large Australian SSc cohort.

**Methods:**

PAH was diagnosed on right heart catheterisation (mPAP >25 and PAWP <15 mmHg at rest). Patients with PH secondary to interstitial lung disease (ILD; defined as abnormal HRCT scan and FVC < 60 %) were excluded. Summary statistics, chi-square tests, univariate and multivariable logistic regression along with post-estimation diagnostics were used to determine the associations of different combinations of risk factors with PAH.

**Results:**

Among 1579 SSc patients, 8.4 % (132 patients) were diagnosed with PAH over a mean (±SD) follow-up of 3.2 (±2.5) years. The incidence of PAH in this cohort was 0.7 % per annum. Of these, 68.9 % had limited disease subtype (lcSSc). In multivariable regression analysis, the presence of anti-centromere antibody (ACA) (OR 1.6, 95 % CI 1.1–2.5, *p* = 0.03), oesphageal stricture (OR 2.0, 95 % CI 1.2–3.3, *p* = 0.006), calcinosis (OR 1.9, 95 % CI 1.2–2.9, *p* = 0.003), sicca symptoms (OR 1.6, 95 % CI 1.1–2.5, *p* = 0.03), mild ILD (OR 2.3, 95 % CI 1.5–3.7, *p* < 0.001) and digital ulcers (OR 1.6, 95 % CI 1.0–2.4, *p* = 0.03) were predictive of PAH. This model had an area under the curve of 0.7 and concordance of 91.8 %. When analysed by disease subtype, the presence of calcinosis (OR 2.2, 95 % CI 1.4–3.7, *p* = 0.01), sicca symptoms (OR 2.6, 95 % CI 1.5–4.6, *p* = 0.001), mild ILD (OR 2.3, 95 % CI 1.4–3.8, *p* = 0.001) and digital ulcers (OR 1.9, 95 % CI 1.2–3.7, *p* = 0.01) were predictive of PAH in lcSSc; and oesophageal stricture (OR 4.4, 95 % CI 1.9–10.5, *p* = 0.001), mild ILD (OR 2.8, 95 % CI 1.2–6.8, *p* = 0.02) and ACA (OR 5.2, 95 % CI 1.8–14.8, *p* = 0.002) were predictive of PAH in dcSSc.

**Conclusions:**

The incidence and prevalence of PAH in this cohort are 0.7 % per annum and 8.4 %, respectively. The clinical-serologic risk factors for PAH differ based on disease subtype. In both subtypes, mild ILD is associated with PAH, suggesting the possibility of common pathogenic mechanisms underlying both of these disease manifestations. This model identifies a subset of patients at an appreciably higher risk of developing PAH, who should be screened and would in future, benefit from preventative therapies.

**Electronic supplementary material:**

The online version of this article (doi:10.1186/s12890-016-0296-z) contains supplementary material, which is available to authorized users.

## Background

Systemic sclerosis (SSc) is a multisystem autoimmune disease characterised by fibrosis of skin and internal organs, vasculopathy and immune dysregulation. It is classified into distinct subtypes based on the degree of skin fibrosis, namely limited (lcSSc) and diffuse cutaneous (dcSSc) subsets. SSc is a complex clinically heterogeneous disease, the underlying aetiology of which is not fully understood. It is presumed to occur in a genetically susceptible host after exposure to an environmental trigger [1]. Currently there are no effective disease modifying agents or cure.

Despite an improvement over the last three decades, morbidity and mortality in SSc remain high. Survival is well below age and gender matched controls, with an overall standardised mortality ratio of 4.08 and a ten-year survival of 75 % in a newly diagnosed patient with diffuse disease [2].

Cardiorespiratory manifestations, namely pulmonary arterial hypertension (PAH) and interstitial lung disease (ILD), remain the leading causes of SSc-related deaths. SSc-PAH, as diagnosed on right heart catheterisation (mPAP >25 and PAWP <15 mmHg at rest), occurs with a prevalence of 8–12 % and is associated with reduced life expectancy [3]. The pathogenesis of SSc-PAH is unknown, but thought to be a combination of endothelial cell dysfunction and an imbalance of endothelial derived vasoactive substances [1].

The difficulty with diagnosing SSc-PAH is that the clinical manifestations are non-specific. Early in the disease process, patients may remain asymptomatic. Later, they may complain of lethargy, fatigue, exertional dyspnoea and/or symptoms of right heart failure, by which time PAH is likely to be moderate to severe, with an average three-year survival of 40–50 % [4]. In addition to reducing the quantity of life, PAH also impacts significantly on the quality of these years of life, reflected by poor patient reported health related quality of life scores [5].

Consequently, annual screening programs aimed at detecting PAH in its early phases have been recommended for all patients with SSc, to enable an earlier diagnosis of PAH and early commencement of aggressive therapy with the goal of prolonging life years and improving quality of life [6]. These screening programs have been shown to be cost effective [7]. In the last decade, with the introduction of potent pulmonary vasodilator therapies, patients’ quality of life and survival have improved [8].

Given the availability and potential of such agents, it is important to identify patients who are at increased risk of developing PAH. Certain hemodynamic characteristics on transthoracic echocardiography (TTE) and reduced diffusing capacity for carbon monoxide corrected for haemoglobin (DLCOc) on pulmonary function testing (PFT) point to the presence of PAH and are used in screening algorithms for SSc-PAH, but no predictive model based specifically on clinical manifestations and antibody status has been reported.

We sought to determine the incidence, prevalence and clinical risk factors for PAH development in a large cohort of Australian patients.

## Methods

### Patients

All patients were enrolled in the Australian Scleroderma Cohort Study (ASCS) and fulfilled either the American College of Rheumatology or Leroy and Medsger criteria for SSc [9, 10]. The ASCS is a multi-center study of risk and prognostic factors for cardiopulmonary outcomes in SSc. It comprises 13 Australian centers and has been approved by the human research ethics committees of each of the participating hospitals (St. Vincent’s Hospital, Melbourne, Royal Adelaide Hospital, Monash Health, Royal Perth Hospital, The Queen Elizabeth Hospital, Sunshine Coast Rheumatology, Prince Charles Hospital, John Hunter Hospital, Royal North Shore Hospital, Royal Prince Alfred Hospital, St George Hospital, Canberra Rheumatology and the Menzies Research Institute Tasmania). All patients provide written informed consent at recruitment.

### Inclusion and exclusion criteria

We included all consecutive adult (>18 years) SSc patients from the ASCS recruited between December 2007 and June 2015. All patients were screened annually for PAH with PFTs and TTE. Any patient with a high risk of PAH (defined as systolic pulmonary arterial pressure (sPAP_TTE_) of at least 40 mmHg and/or DLCOc less than 50 % predicted with forced vital capacity (FVC) of more than 85 % predicted (without adequate explanation on high-resolution computer tomography (HRCT) of the lung or ventilation-perfusion (V/Q) scan of lung or both) underwent right heart catheterisation (RHC).

As per the current World Health Organization (WHO) classification [11], patients were classified as having SSc-PAH (WHO Group 1) if RHC-determined mean pulmonary arterial pressure (mPAP) was at least 25 mmHg with a pulmonary artery wedge pressure (PAWP) of ≤15 mmHg with no more than mild ILD on HRCT and a FVC of more than 60 % predicted. A patient was classified as having PAH from the date of their first RHC confirming PAH.

Patients were excluded if they had WHO Group 2 or 3 pulmonary hypertension or group 1 pulmonary arterial hypertension but with co-existing moderate-severe ILD with a FVC <60 % and an abnormal HRCT (see below).

Patients were divided into two categories based on their diagnosis of PAH: SSc patients with PAH and SSc patients without PAH.

### Data collection

Patient demographics, clinical variables, cardiac and pulmonary assessments were prospectively recorded. All physical examination and investigation data were collected within one month of the first RHC, before starting pulmonary vasodilator therapy. TTE was performed according to standardised procedures only at tertiary centers. Pulmonary involvement was assessed by clinical examination and PFT and HRCT if ILD was suspected. All disease features were defined as present ever from time of diagnosis. Sicca symptoms were defined as dry eyes or dry mouth for more than four annual reviews. ILD was defined based on characteristic interstitial changes on HRCT which were performed and interpreted by a radiologist at the tertiary hospital with a specialised SSc clinic. Each HRCT scan was graded based on total extent of lung involvement into limited (<20 % involved) and extensive (>20 %). Those where the disease extent was unclear were classified as ‘indeterminate’. All patients with indeterminate or extensive ILD were excluded from this study. Myocardial disease was defined on endomyocardial biopsy or as the presence of conduction defects, arrhythmia, right ventricular or left ventricular dysfunction in the absence of other causes. Pericardial effusions were defined on echocardiogram other than a small non-significant pericardial effusion. Renal crisis was defined on a combination of any two of the following three criteria, which included new onset severe hypertension (≥180 mmHg systolic and/or ≥ 100 mmHg) without an alternate aetiology, microangiopathic hemolytic anemia or rising creatinine. Gastric antral vascular ectasia (GAVE), reflux oesophagitis and oesophageal stricture were defined on endoscopy. Bowel dysmotility was defined based on barium and nuclear medicine studies, antibiotic response or characteristic symptoms.

At each annual visit, patients completed a health related quality of life form (Medical Outcomes Study Short form-36 (SF-36 form)) which is a validated tool for measuring health related quality of life (HRQoL) in patients with rheumatic conditions including SSc [12]. The classification of patients into lcSSc and dcSSc was confirmed by reviewing their peak modified Rodnan skin scores which has been shown to be a valid and reliable method for differentiating between SSc disease subtypes [13].

### Statistical analysis

Data are presented as mean ± standard deviation for continuous variables and as number (percentage) for categorical variables. The difference in frequency was determined by using chi-square and Fisher’s exact tests. The predictive accuracy of the models is presented as sensitivity, specificity, positive (PPV) and negative predictive values (NPV), with 95 % confidence intervals (CI). Negative predictive accuracy requires the exclusion of PAH on RHC; however it would not be feasible for all patients to undergo such an invasive procedure. Thus, we defined no PAH as those who do not fulfill ‘at risk of PAH criteria’, defined as sPAP >/=40 mmHg and/or DLCOc <50 % and FVC >85 % predicted. Summary statistics, univariable and multivariable logistic regression were performed to determine the predictors of PAH. Statistically significant variables (defined by a *p*-value of <0.05) and or variables previously reported in the literature to be predictors of PAH where included in the multivariable analysis. Post-estimation diagnostics were used to determine the associations of different combinations of risk factors with PAH. Area under the curve and goodness of fit and concordance were used to assess the models. A two-tailed *p* value of not more than 0.05 was considered statistically significant. All statistical analyses were performed using STATA 14.0 (StataCorp LP, College Station, TX, USA).

## Results

### Patient characteristics

Among 1579 SSc patients in the ASCS, 8.4 % (132 patients) were diagnosed with WHO Group 1 PAH. The incidence of PAH in this cohort was 0.7 % per annum. PAH occurred more frequently in women and in the limited subtype, but this did not reach statistical significance. At PAH diagnosis, the mean (±SD) age was 62 (±10.5) years and the disease duration from the first non-Raynaud’s manifestation was 14 (±12) years. The mean (±SD) follow-up of 3.2 (±2.5) years from cohort entry. Disease duration at PAH diagnosis was not significantly different between disease subtypes [lcSSc 15.3 (±12.4) years vs dcSSc 12.8 (±11.3) years, *p* = 0.40)]. There was no significant difference in the frequency of anti-centromere pattern antinuclear antibody (ACA; determined by immunofluorescence) or anti-phospholipid antibodies (APLA; determined by ELISA) in patients with and without PAH (Table 1). Anti-topoisomerase I antibody (Scl-70; determined by ELISA) was more common in those without PAH (14.1 %,) than in those with PAH (7.4 %), *p* = 0.04. Clinical manifestations in those with and without PAH are summarised in Table 1.

**Table 1:** Characteristics of patients with and without SSc-PAH (*n* = 1579)

	PAH	No PAH	*p*
	*n* = 132	*n* = 1447	
	mean ± SD or %	mean ± SD or %	
Female	84.9 % 112	86.7 % 1254	0.56
Age at recruitment, years	62.7 ± 10.3	56.6 ± 12.8	<0.001
Race			
Caucasian Asian Aboriginal-Islander Hispanic Other	112 (84.9 %) 6 (4.6 %) 1 (0.8 %) 1 (0.8 %) 0	1234 (85.3 %) 61 (4.2 %) 15 (1.1 %) 9 (0.6 %) 14 (0.9 %)	0.89
Follow-up duration Total 3.2 (±2.5)	3.5 (±2.4)	3.1 (±2.5)	0.13
Age at PAH diagnosis	62.3 (±10.5)	n/a	
Disease duration^a^ at recruitment, years Total: 11.1 (±10.2)	14.4 ± 12.1	10.8 ± 9.9	0.002
Disease subtype			
Limited Diffuse MCTD	91 (68.9 %) 30 (22.7 %) 7 (5.3 %)	959 (66.3 %) 353 (24.4 %) 70 (4.8 %)	0.82
Anti-centromere pattern ANA	63 (51.6 %)	584 (43.6 %)	0.10
Scl 70 positive	9 (7.4 %)	189 (14.1 %)	0.04
Antiphospholipid antibody	33 (27.1 %)	292 (21.8 %)	0.12
RNA polymerase III positive	8 (11.4 %)	100 (12.4 %)	0.57
SSc associated Disease manifestations			
GAVE Reflux oesphagitis Oesphageal stricture Oesphageal dysmotility Bowel dysmotility Anal incontinence Myocardial Pericardial effusion Renal crisis Myositis Synovitis Joint contractures Calcinosis Digital Ulcers Sicca symptoms ILD Telangiectasia Rodnan skin score (highest ever)	16 (12.2 %) 65 (49.2 %) 29 (21.9 %) 16 (12.1 %) 41 (31.1 %) 41 (31.1 %) 10 (7.6 %) 24 (18.2 %) 2 (1.5 %) 0 27 (20.5 %) 58 (43.9 %) 70 (53.0 %) 72 (54.6 %) 84 (63.6 %) 51 (38.6 %) 114 (86.4 %) 13.4 ± 9.6	128 (8.9 %) 688 (47.6 %) 142 (9.8 %) 127 (8.8 %) 338 (23.4 %) 340 (23.5 %) 97 (6.7 %) 72 (4.9 %) 37 (2.6 %) 29 (2.0 %) 298 (20.6 %) 478 (33.0 %) 473 (32.7 %) 592 (40.9 %) 730 (50.5 %) 353 (24.4 %) 1119 (77.3 %) 10.9 ± 9.5	0.21 0.71 <0.0001 0.20 0.05 <0.0001 0.51 <0.001 0.46 0.10 0.86 <0.001 <0.001 0.01 <0.001 <0.001 0.01 0.01
SF 36 score			
Physical functioning Role – physical Bodily pain General health Vitality Social functioning Role – emotional Mental health	35.2 ± 21.2 30.0 ± 34.7 66.2 ± 10.2 32.6 ± 16.9 43.5 ± 17.7 62.6 ± 25.0 55.3 ± 37.8 61.8 ± 21.7	57.9 ± 56.0 48.3 ± 39.9 46.3 ± 26.5 46.8 ± 21.4 49.3 ± 23.6 70.6 ± 24.9 67.8 ± 35.7 68.3 ± 19.4	<0.001 <0.001 <0.001 <0.001 0.03 0.01 0.01 0.01

*Abbreviations*: *GAVE* gastric antral vascular ectasia, *ILD* interstitial lung disease, *PAH* pulmonary arterial hypertension

^a^disease duration from first non-Raynaud manifestation

Patients with SSc-PAH had significantly lower health related quality of life scores in all domains of the SF-36 form compared with SSc patients without PAH (Table 1).

Features found more commonly in patients with PAH included gastrointestinal manifestations such as oesphageal stricture, bowel dysmotility and anal incontinence, pericardial effusion, joint contractures, calcinosis, digital ulcers, sicca symptoms, mild ILD, higher modified Rodnan skin score and telangiectasia (all *p* < 0.01)

### Clinical variables predictive of developing SSc-PAH

In univariable analysis, clinical variables associated with the development of PAH included gastrointestinal manifestations such as oesphageal stricture, bowel dysmotility, anal incontinence, joint contractures, calcinosis, digital ulcers, sicca symptoms, mild ILD, telangiectasia, disease duration at recruitment and the absence of anti-topoisomerase I (Scl-70) antibody (Table 2).

**Table 2:** Univariable logistic regression analysis

Predictors of PAH	OR (95 % CI)	*p*
GAVE	1.42 (0.8–2.5)	0.21
Reflux oesphagitis	1.07 (0.8–1.5)	0.71
Oesphageal stricture	2.59 (1.7–4.0)	<0.001
Oesphageal dysmotility	1.43 (0.8–2.5)	0.20
Bowel dysmotility	1.47 (1.0–2.2)	0.05
Anal incontinence	1.66 (1.1–2.5)	0.01
Myocardial	1.14 (0.6–2.2)	0.70
Pericardial effusion	4.44 (2.7–7.4)	<0.001
Renal crisis	0.59 (0.2–2.5)	0.47
Synovitis	0.96 (0.6–1.5)	0.86
Joint contractures	1.79 (1.2–2.6)	0.002
Calcinosis	2.65 (1.8–3.9)	<0.001
Digital Ulcers	1.85 (1.3–2.7)	0.001
Sicca symptoms	2.23 (1.5–3.4)	<0.001
ILD	1.95 (1.4–2.8)	<0.001
Telangiectasia	3.02 (1.5–6.0)	0.002
Disease duration at recruitment	1.03 (1.01–1.05)	<0.001
Limited systemic sclerosis	1.13 (0.8–1.7)	0.54
Autoantibodies		
Anti-centromere pattern	1.36 (0.9–1.9)	0.11
Scl 70 + ve	0.49 (0.2–0.9)	0.05
RNA polymerase III + ve	0.89 (0.4–1.9)	0.78

*Abbreviations*: *GAVE* gastric antral vascular ectasia, *ILD* interstitial lung disease, *PAH* pulmonary arterial hypertension

Using multivariable logistic regression together with post-estimation diagnostics, we were able to determine the associations of different combinations of risk factors with the development of SSc-PAH. We created a six variable model for the prediction of PAH in patients with SSc. These clinical variables and their associated odds ratios for development of PAH are presented in Table 3. ACA was associated with a 1.6-fold increased odds of developing SSc-PAH, oesphageal stricture with 2-fold increased odds, calcinosis with a 1.9-fold increased odds, digital ulcers with a 1.6-fold increased odds, mild ILD with a 2.3-fold increased odds and sicca symptoms with a 1.6-fold increased odds of PAH. This six variable model had an area under the curve of 0.70, goodness of fit *p* = 0.28 and concordance of 91.8 %. The sensitivity, specificity, PPV and NPV of this model are 100 % (95 % CI 99.7–100 %), 99.9 % (95 % CI 99.3–99.9 %), 100 % (95 % CI 15.8–100 %) and 91.8 % (95 % CI 90.3–93.1 %), respectively. While only 2 patients with PAH had all six variables present, the presence of any four or more of the six risk factors gave predictive properties similar to those presented for the complete model (Additional file 1).

**Table 3:** Multivariable analysis of predictors of PAH in SSc

Predictors of PAH	OR (95 % CI)	*p*
Presence of anticentromere antibody	1.63 (1.1–2.5)	0.03
Oesphageal stricture	2.00 (1.2–3.3)	0.01
Calcinosis	1.88 (1.2–2.9)	0.01
Digital Ulcers	1.56 (1.0–2.4)	0.03
ILD	2.34 (1.5–3.7)	<0.001
Sicca	1.62 (1.1–2.5)	0.03

*Abbreviations*: *ILD* interstitial lung disease, *PAH* pulmonary arterial hypertension

### Clinical variables predictive of SSc-PAH according to disease subtype

By using multivariable logistic regression along with post-estimation diagnostics and analysing by disease subtype, we created PAH predictive models for each disease subtype.

For lcSSc, we created a four clinical variable model for the prediction of PAH (Table 4). Calcinosis was associated with a 2.2-fold increased odds of PAH, sicca symptoms with a 2.6-fold increased odds, mild ILD with a 2.3-fold increased odds and digital ulcers with a 1.9-fold increased odds of developing PAH. This model has an area under the curve of 0.73 with a goodness of fit *p* = 0.96 and concordance of 89.6 %. The sensitivity, specificity, PPV and NPV of this model are 7.7 % (95 % CI 3.2–15.2 %), 97.4 % (95 % CI 96.2–98.3 %), 21.9 % (95 % CI 9.3–40.0 %) and 91.7 % (95 % CI 89.9–93.4 %). Thirty-two of 91 (35.2 %) patients with lcSSc and PAH had all 4 variables.

**Table 4:** Multivariable analysis of predictors of PAH in lcSSc

Predictors of PAH	OR (95 % CI)	*p*
Calcinosis	2.24 (1.4–3.7)	0.001
Sicca	2.63 (1.5–4.6)	0.001
ILD	2.31 (1.4–3.8)	0.001
Digital Ulcers	1.85 (1.2–3.7)	0.01

*Abbreviations*: *ILD* interstitial lung disease, *lcSSc* limited cutaneous systemic sclerosis, *PAH* pulmonary arterial hypertension

For dcSSc, we created a three clinical variable model for the prediction of PAH (Table 5). Oesphageal stricture was associated with a 4.4-fold increased odds of PAH, mild ILD with a 2.8-fold increased odds and the presence of ACA was associated with a 5.2-fold increased odds of developing PAH. This model has an area under the curve of 0.73, goodness of fit *p* = 0.79 and concordance of 92.7 %. The sensitivity, specificity, PPV and NPV of this model are 6.7 % (95 % CI 0.8–22.1 %), 100 % (95 % CI 99–100 %), 100 % (95 % CI 15.8–100 %), and 92.7 % (95 % CI 89.6–95.1 %). However, only 2 of 30 (7 %) patients with dcSSc and PAH had all 3 variables.

**Table 5:** Multivariable analysis of predictors of PAH in dcSSc

Predictors of PAH	OR (95 % CI)	*p*
Oesphageal stricture	4.42 (1.9–10.5)	0.001
ILD	2.83 (1.2–6.8)	0.02
ACA	5.16 (1.8–14.8)	0.002

*Abbreviations*: *dcSSc* diffuse cutaneous systemic sclerosis, *ILD* interstitial lung disease, *PAH* pulmonary arterial hypertension, *ACA* anticentromere antibody

## Discussion

In this cohort of Australian SSc patients, PAH occurred with a prevalence of 8.4 %, more frequently in women (84.9 %) and those with limited disease subtype (68.9 %) and longer disease duration [14.4 (±12) years], which is consistent with reports in the literature [14, 15]. Additionally in our cohort, patients’ mean age at PAH diagnosis was 62.7 (±10.3) years. The literature suggests a two fold increased risk of PAH in those diagnosed with SSc over the age of 60 years compared with those under the age of 60 years [16]. This may reflect progressive vasculopathy related to longer disease duration and increasing age. The high frequency of PAH in women has been hypothesised to be a consequence of decreasing oestrogen levels and loss of their protective vascular effects in the postmenopausal state [17]. This has led to the investigation of hormone replacement therapy in the prevention and treatment of PAH [18].

It is important to note that in the model for all SSc patients, irrespective of disease subtype, the presence of any four or more of the six variables listed is associated with a substantially increased risk of developing PAH, and that patients need not have all of the six variables present to be deemed ‘at risk’.

Historically, SSc-PAH was thought to predominantly affect patients with lcSSc [19]. However, our study and others have shown that it may also occur in patients with dcSSc [20]. Although the literature suggests that increasing disease duration is associated with increasing risk of PAH, as in our cohort, other studies have shown that PAH can occur early, in about 25–50 % of SSc patients within the first five years of the first SSc-related non Raynaud’s manifestation [21, 22].

Vasculopathy is a characteristic component of SSc. Therefore it is not surprising that obliterative vasculopathy of the pulmonary microvasculature, as occurs in PAH, would be associated with vasculopathy of the peripheral microvasculature resulting in severe Raynaud’s phenomenon, digital ulcers and digital amputation. In keeping with the literature [23], we showed a strong association between digital ulcers and PAH in our SSc model and also in the lcSSc model. However, this association was not seen in the dcSSc model. The reason for this is unclear, but may relate to worse vasculopathy in those with lcSSc due to their longer disease duration.

Similarly, telangiectasiae are vascular lesions composed of vasodilated post-capillary venules without evidence of neovascularisation or inflammation, that have been reported as a risk factor for the development of SSc-PAH [24]. In our study, this association was only significant in univariable analysis. This may be due to a specific pattern or size of telangiectasia, which is not recorded in our data set. The pathogenesis is thought to be the consequence of an aberrant attempt at increasing blood perfusion to hypoxic tissues due to the loss of normal circulation. Thus, telangiectasiae have been hypothesised to be markers of ongoing vascular injury and failed vascular repair [24]. As the disease duration of both patients with and without PAH in our cohort was quite long [11.1 (±10) years] and the presence and frequency of telangiectasiae is known to be strongly associated with disease duration, this may explain why we did not show a significant association with telangiectasia in those with PAH.

Calcinosis is the deposition of calcium in the skin and subcutaneous tissues occurring in about 22 % of SSc patients. Local trauma, chronic inflammation and vascular hypoxia secondary to vasculopathy of the dermal and subcutaneous microvasculature with subsequent tissue hypoxia have been hypothesised to be the main underlying pathogenic mechanisms causing an increased calcium influx into cells [25]. Hence, the association between calcinosis and PAH as we and others have shown is plausible [25].

Chronic, silent micro-aspiration, usually as a result of untreated gastro-oesphageal reflux disease (GORD), is a cause of significant morbidity and mortality. Chronic, untreated GORD and micro-aspiration can result in oesphageal strictures. The aetiology behind the strong associated between oesphageal strictures and SSc-PAH in our study is unclear. We know from the lung transplant literature that micro-aspiration is a significant risk factor in the development of bronchiolitis obliterans and subsequent transplant failure [26]. So much so that it is recommended that all patients be screened with oesphageal pH manometry prior to being considered for transplantation [27]. We postulate that this may be due to an inflammatory cytokine milieu caused by micro-aspiration and GORD initiating a cascade of inflammation, remodelling and fibrosis within the pulmonary vasculature leading to PAH. This microvascular reaction leading to PAH may be the same reactive process occurring in the microvascular bed, causing damage to the oesophageal muscle or neurological plexus resulting in the motility disorder causing GORD. Additionally, overnight hypoxaemia secondary to aspiration from GORD may contribute as a driver of PAH.

Furthermore, GORD and chronic, silent micro-aspiration have been implicated in the pathogenesis and natural history of ILD, with GORD occurring in ILD with a prevalence of 90 % [28]. These are areas where further research is required to elucidate key pathophysiologic relationships. Additionally, the routine use of proton pump inhibitors in all patients with SSc, not only those symptomatic of GORD (given the poor predictive power of patient reported symptoms) should be considered.

SSc-ILD is a frequent manifestation of SSc, occurring in up to 75 % of patients overall but leading to end stage respiratory failure in only a proportion [29]. The pathogenesis is not well understood, but is thought to be the consequence of a combination of inflammation, fibrosis and cell proliferation leading to increased production of extra-cellular matrix proteins by fibroblasts [29]. SSc-ILD can occur in isolation or in combination with PAH, where PAH is a direct consequence of severe ILD (Group 3 PH which we excluded in this study) or where the two pathologies co-exist (PAH and mild ILD not sufficient enough to cause PH). The co-existence PAH and mild ILD occurs in about 25 % of patients with SSc-ILD [29]. The presence of anti-phospholipid antibodies, particularly anti-cardiolipin antibodies, is a risk factor for developing co-existent mild ILD and PAH [30]. In this study, we excluded patients with severe ILD (FVC < 60 % and abnormal HRCT) in whom pulmonary hypertension may have occurred secondary to ILD. However, we found that mild ILD, which in itself was not significant enough to cause PAH, was a risk factor for the subsequent development of PAH, perhaps suggesting a shared underlying pathogenic mechanism.

Sicca symptoms are common in SSc and are explained by fibrosis of the exocrine glands. They are significant contributors to poor health related quality of life. The association between sicca symptoms and PAH has been investigated in studies before with conflicting results [31, 32] but these studies had low patient numbers and may have been underpowered. Sicca symptoms, particularly xerostomia, are associated with varying degrees of oesphageal motor dysfunction regardless of patient reported dysphagia [33]. Accordingly, we propose that this association between sicca symptoms and PAH may be a consequence of micro-aspiration secondary to oesphageal dysmotlility.

Certain autoantibodies in SSc are associated with a higher risk of developing SSc-PAH. These include ACA [34], anti-phospholipid antibodies [35], anti-U1-ribonucleoprotein antibodies [36], nucleolar pattern of antinuclear antibodies [36], anti-endothelial antibodies [37] and anti-4-sulfated N-acetyllactosamine antibodies [38]. Testing for these last two autoantibodies is not yet clinically available. In our study, the presence of ACA and the absence of anti-topoisomerase I (Scl-70) antibody were associated with a higher risk of PAH. The association between PAH and the absence of anti-topoisomerase I (Scl-70) antibody has been reported previously [23]. Of interest to us was the strong association between the presence of ACA and the development of PAH in dcSSc. This association was not shown in the lcSSc model, perhaps because of the high prevalence of ACA in almost all patients with lcSSc. This is a novel finding and we propose that this may be due to a more ‘CREST’ (calcinosis, Raynaud’s phenomenon, oesphageal dysmolity, sclerodactyly and telangiectasia) like profile in these dcSSc patients.

Given the significant burden of disease associated with SSc-PAH, it is not surprising that patients have worse health related quality of life scores in all domains in the SF-36 form. This finding is consistent with the literature [5] and the introduction of vasodilator therapy has shown improvement in patient’s quality of life [39].

As presented, we propose predictive models for SSc-PAH based solely on clinical manifestations and autoantibody status, which to our knowledge, has not before been reported in the literature. Whilst these models are not screening or diagnostic models, they can aid in identifying patients who are at an appreciably higher risk of developing SSc-PAH who should be monitored and undergo annual screening for PAH. These patients are also potential candidates for PAH preventive therapy once this becomes available in the future.

We recognise that there are limitations to our study. Our study was cross-sectional in nature and undertaken in a predominantly ‘prevalent’ SSc cohort. Future studies evaluating predictors of PAH in a purely incident SSc cohort would be highly informative. In addition, as RHC was not performed in every patient at every visit, it is possible that some patients who did not undergo RHC were misclassified as not having PAH. However, RHC was performed in almost all patients ‘at risk’ of PAH based on screening echocardiography and the likelihood of missed cases of PAH is therefore very low.

## Conclusions

Pulmonary arterial hypertension is the leading causes of SSc-related death. With the advent of potent pulmonary vasodilators and their associated impact on patients’ symptoms, quality of life and survival, it is imperative that we begin to detect PAH in its early stages to optimise care. We have introduced clinico-serologic models for prediction of PAH. Interestingly, the clinical-serologic risk factors for PAH differ based on disease subtype. In both subtypes, mild ILD is associated with PAH, suggesting possible common pathogenic mechanisms underlying both of these disease manifestations. Whilst not a screening or diagnostic model, these models help to identify a subset of patients at an appreciably higher risk of developing PAH, who should be screened and would in future benefit from preventative therapies when these become available.

## Additional file

Additional file 1: Comparison of the predictive models based on variable number. (DOCX 126 kb)

## Abbreviations

dcSSc: Diffuse systemic sclerosis
ILD: Interstitial lung disease
lcSSc: Limited systemic sclerosis
PAH: Pulmonary arterial hypertension
SSc: Systemic sclerosis (scleroderma)
