# Supplementary material for: Risk factors for development of pulmonary arterial hypertension in Australian systemic sclerosis patients: results from a large multicenter cohort study
Source: BMC Pulm Med. 2016 Sep 27;16:134. doi: 10.1186/s12890-016-0296-z (PMC5039932; doi:10.1186/s12890-016-0296-z)
Supplement: Additional file 1: — Comparison of the predictive models based on variable number. (DOCX 126 kb) [file 12890_2016_296_MOESM1_ESM.docx]

**Comparison of the predictive models based on variable number**

**Complete 6-variable model**

|  | **PAH** | **No PAH** | ***p* value** |
| --- | --- | --- | --- |
|  | **n = 132** | **n = 1447** |  |
| All 6 variables | 2 | 2 | <0.003 |
| None of the six variables | 128 | 1445 |  |

|  | **%** | **95% CI** |
| --- | --- | --- |
| Prevalence | 8.2 | 6.9-9.7 |
| Sensitivity | 1.5 | 0.2-5.5 |
| Specificity | 99.9 | 99.5-100 |
| PPV | 50 | 6.7-93.2 |
| NPV | 91.9 | 90.4-93.2 |
| LR (+) | 11.1 | 1.6-78.4 |
| LR (-) | 0.9 | 0.9-1.0 |
| Concordance | 91.8% | |
| Area under the curve | 0.700 | |

**Comparison with the 4-variable models**

**Model One**

Variables included ACA, oesphageal stricture, digital ulcer, ILD

|  | **PAH** | **No PAH** | ***p* value** |
| --- | --- | --- | --- |
|  | **n = 132** | **n = 1447** |  |
| All 4 variables | 2 | 2 | <0.003 |
| None of the 4 variables | 130 | 1445 |  |

|  | **%** | **95% CI** |
| --- | --- | --- |
| Prevalence | 8.2 | 6.9-9.7 |
| Sensitivity | 1.5 | 0.2-5.5 |
| Specificity | 99.9 | 99.5-100 |
| PPV | 50 | 6.7-93.2 |
| NPV | 91.9 | 90.4-93.2 |
| LR (+) | 11.1 | 1.6-78.4 |
| LR (-) | 0.9 | 0.9-1.0 |
| Concordance | 91.6% | |
| Area under the curve | 0.65 | |

**Model Two**

Variables included ACA, digital ulcer, ILD, calcinosis

|  | **PAH** | **No PAH** | ***p* value** |
| --- | --- | --- | --- |
|  | **n = 132** | **n = 1447** |  |
| All 4 variables | 6 | 12 | <0.001 |
| None of the 4 variables | 126 | 1435 |  |

|  | **%** | **95% CI** |
| --- | --- | --- |
| Prevalence | 8.3 | 7.0-9.6 |
| Sensitivity | 4.5 | 1.7-9.6 |
| Specificity | 99.2 | 98.6-99.6 |
| PPV | 33 | 13.3-59 |
| NPV | 92 | 90.5-93.3 |
| LR (+) | 5.5 | 2.1-14.5 |
| LR (-) | 0.9 | 0.9-1.0 |
| Concordance | 91.3% | |
| Area under the curve | 0.67 | |

**Model Three**

Variables included ACA, ILD, calcinosis, sicca

|  | **PAH** | **No PAH** | ***p* value** |
| --- | --- | --- | --- |
|  | **n = 132** | **n = 1447** |  |
| All 4 variables | 9 | 12 | <0.001 |
| None of the 4 variables | 123 | 1435 |  |

|  | **%** | **95% CI** |
| --- | --- | --- |
| Prevalence | 8.4 | 7.0-9.8 |
| Sensitivity | 6.8 | 3.2-12.5 |
| Specificity | 99.2 | 98.6-99.6 |
| PPV | 42.9 | 21.8-66 |
| NPV | 92 | 90.7-93.4 |
| LR (+) | 8.2 | 3.5-19.2 |
| LR (-) | 0.9 | 0.9-1.0 |
| Concordance | 91.5% | |
| Area under the curve | 0.67 | |

**Model Four**

Variables included stricture, digital ulcer, ILD, calcinosis

|  | **PAH** | **No PAH** | ***p* value** |
| --- | --- | --- | --- |
|  | **n = 132** | **n = 1447** |  |
| All 4 variables | 7 | 12 | <0.001 |
| None of the 4 variables | 125 | 1435 |  |

|  | **%** | **95% CI** |
| --- | --- | --- |
| Prevalence | 8.4 | 7.0-9.8 |
| Sensitivity | 5.3 | 2.2-10.6 |
| Specificity | 99.2 | 98.6-99.6 |
| PPV | 36.8 | 16.3-61.6 |
| NPV | 92 | 90.5-93.3 |
| LR (+) | 8.2 | 3.5-19.2 |
| LR (-) | 0.9 | 0.9-1.0 |
| Concordance | 91.3% | |
| Area under the curve | 0.67 | |

**Model Five**

Variables included stricture, digital ulcer, sicca, calcinosis

|  | **PAH** | **No PAH** | ***p* value** |
| --- | --- | --- | --- |
|  | **n = 132** | **n = 1447** |  |
| All 4 variables | 12 | 37 | <0.001 |
| None of the 4 variables | 120 | 1410 |  |

|  | **%** | **95% CI** |
| --- | --- | --- |
| Prevalence | 8.4 | 7.0-9.8 |
| Sensitivity | 9.1 | 4.8-15.3 |
| Specificity | 97.4 | 96.5-98.2 |
| PPV | 24.5 | 13.3-38.9 |
| NPV | 92.2 | 90.7-93.5 |
| LR (+) | 3.6 | 1.9-7.4 |
| LR (-) | 0.9 | 0.9-1.0 |
| Concordance | 90.1% | |
| Area under the curve | 0.67 | |

**Model Six**

Variables included ILD, calcinosis, sicca, stricture

|  | **PAH** | **No PAH** | ***p* value** |
| --- | --- | --- | --- |
|  | **n = 132** | **n = 1447** |  |
| All 4 variables | 9 | 13 | <0.001 |
| None of the 4 variables | 123 | 1434 |  |

|  | **%** | **95% CI** |
| --- | --- | --- |
| Prevalence | 8.4 | 7.0-9.8 |
| Sensitivity | 6.8 | 3.2-12.5 |
| Specificity | 99.1 | 98.5-99.5 |
| PPV | 40.9 | 20.7-63.6 |
| NPV | 92.1 | 90.6-93.5 |
| LR (+) | 7.6 | 3.3-17.4 |
| LR (-) | 0.9 | 0.9-1.0 |
| Concordance | 91.4% | |
| Area under the curve | 0.69 | |

**Model Seven**

Variables included ILD, sicca, digital ulcer , calcinosis

|  | **PAH** | **No PAH** | ***p* value** |
| --- | --- | --- | --- |
|  | **n = 132** | **n = 1447** |  |
| All 4 variables | 15 | 62 | <0.001 |
| None of the 4 variables | 117 | 1385 |  |

|  | **%** | **95% CI** |
| --- | --- | --- |
| Prevalence | 8.4 | 7.0-9.8 |
| Sensitivity | 11.4 | 6.5-18 |
| Specificity | 95.7 | 94.5-96.7 |
| PPV | 19.5 | 11.3-30.1 |
| NPV | 92.2 | 90.7-93.5 |
| LR (+) | 2.6 | 1.6-4.5 |
| LR (-) | 0.9 | 0.9-1.0 |
| Concordance | 88.7% | |
| Area under the curve | 0.66 | |

**Model Eight**

Variables included ILD, digital ulcer, sicca, stricture

|  | **PAH** | **No PAH** | ***p* value** |
| --- | --- | --- | --- |
|  | **n = 132** | **n = 1447** |  |
| All 4 variables | 8 | 11 | <0.001 |
| None of the 4 variables | 124 | 1436 |  |

|  | **%** | **95% CI** |
| --- | --- | --- |
| Prevalence | 8.4 | 7.0-9.8 |
| Sensitivity | 6.1 | 2.7-11.6 |
| Specificity | 99.2 | 98.6-99.6 |
| PPV | 42.1 | 20.3-66.5 |
| NPV | 92.1 | 90.6-93.3 |
| LR (+) | 7.9 | 3.3-19.5 |
| LR (-) | 0.9 | 0.9-1.0 |
| Concordance | 91.5% | |
| Area under the curve | 0.67 | |

**Model Nine**

Variables included ACA, ILD, sicca, digital ulcer

|  | **PAH** | **No PAH** | ***p* value** |
| --- | --- | --- | --- |
|  | **n = 132** | **n = 1447** |  |
| All 4 variables | 4 | 11 | 0.01 |
| None of the 4 variables | 128 | 1436 |  |

|  | **%** | **95% CI** |
| --- | --- | --- |
| Prevalence | 8.4 | 7.0-9.8 |
| Sensitivity | 3.0 | 0.8-¶.6 |
| Specificity | 99.2 | 98.6-99.6 |
| PPV | 26.7 | 7.8-55.1 |
| NPV | 91.9 | 90.3-93.1 |
| LR (+) | 3.9 | 1.3-12.3 |
| LR (-) | 0.9 | 0.9-1.0 |
| Concordance | 91.2% | |
| Area under the curve | 0.67 | |

**Model Ten - done**

Variables included ACA, sicca, digital ulcer, calcinosis

|  | **PAH** | **No PAH** | ***p* value** |
| --- | --- | --- | --- |
|  | **n = 132** | **n = 1447** |  |
| All 4 variables | 19 | 79 | <0.001 |
| None of the 4 variables | 113 | 1368 |  |

|  | **%** | **95% CI** |
| --- | --- | --- |
| Prevalence | 8.4 | 7.0-9.8 |
| Sensitivity | 14.4 | 8.9-21.6 |
| Specificity | 94.5 | 93.2-95.7 |
| PPV | 19.4 | 12.1-28.6 |
| NPV | 92.4 | 90.9-93.7 |
| LR (+) | 2.9 | 1.7-4.9 |
| LR (-) | 0.9 | 0.9-1.0 |
| Concordance | 87.8% | |
| Area under the curve | 0.67 | |

**Model Eleven**

Variables included digital ulcer, sicca, stricture, ACA

|  | **PAH** | **No PAH** | ***p* value** |
| --- | --- | --- | --- |
|  | **n = 132** | **n = 1447** |  |
| All 4 variables | 10 | 23 | <0.001 |
| None of the 4 variables | 122 | 1424 |  |

|  | **%** | **95% CI** |
| --- | --- | --- |
| Prevalence | 8.4 | 7.0-9.8 |
| Sensitivity | 7.6 | 3.7-13.5 |
| Specificity | 98.4 | 97.6-99 |
| PPV | 30.3 | 15.6-48.7 |
| NPV | 92.1 | 90.7-93.4 |
| LR (+) | 2.9 | 1.7-4.9 |
| LR (-) | 0.9 | 0.9-1.0 |
| Concordance | 90.2% | |
| Area under the curve | 0.65 | |

**Model Twelve**

Variables included ACA, stricture, ILD, sicca

|  | **PAH** | **No PAH** | ***p* value** |
| --- | --- | --- | --- |
|  | **n = 132** | **n = 1447** |  |
| All 4 variables | 4 | 4 | <0.001 |
| None of the 4 variables | 128 | 1443 |  |

|  | **%** | **95% CI** |
| --- | --- | --- |
| Prevalence | 8.4 | 7.0-9.8 |
| Sensitivity | 3.0 | 0.8-7.6 |
| Specificity | 99.7 | 99.3-99.9 |
| PPV | 50 | 15.7-84.3 |
| NPV | 91.9 | 90.4-93.2 |
| LR (+) | 11 | 2.8-43.3 |
| LR (-) | 0.9 | 0.9-1.0 |
| Concordance | 91.6% | |
| Area under the curve | 0.67 | |

**Model Thirteen**

Variables included digital ulcer, calcinosis, ACA, stricture

|  | **PAH** | **No PAH** | ***p* value** |
| --- | --- | --- | --- |
|  | **n = 132** | **n = 1447** |  |
| All 4 variables | 8 | 26 | 0.001 |
| None of the 4 variables | 124 | 1421 |  |

|  | **%** | **95% CI** |
| --- | --- | --- |
| Prevalence | 8.4 | 7.0-9.8 |
| Sensitivity | 6.1 | 2.7-11.6 |
| Specificity | 98.2 | 97.4-98.8 |
| PPV | 23.5 | 10.7-41.2 |
| NPV | 92 | 90.5- 93.3 |
| LR (+) | 3.4 | 1.6-7.3 |
| LR (-) | 0.9 | 0.9-1.0 |
| Concordance | 91.6% | |
| Area under the curve | 0.67 | |

**Model Fourteen**

Variables included calcinosis, ILD, stricture, ACA

|  | **PAH** | **No PAH** | ***p* value** |
| --- | --- | --- | --- |
|  | **n = 132** | **n = 1447** |  |
| All 4 variables | 4 | 2 | <0.001 |
| None of the 4 variables | 128 | 1445 |  |

|  | **%** | **95% CI** |
| --- | --- | --- |
| Prevalence | 8.4 | 7.0-9.8 |
| Sensitivity | 3.0 | 0.8-7.6 |
| Specificity | 99.9 | 99.5-100 |
| PPV | 66.7 | 22.3-95.7 |
| NPV | 91.9 | 90.4- 93.2 |
| LR (+) | 21.9 | 4.1-119 |
| LR (-) | 0.9 | 0.9-1.0 |
| Concordance | 91.8% | |
| Area under the curve | 0.67 | |

**Model Fifteen**

Variables included sicca, calcinosis, stricture, ACA

|  | **PAH** | **No PAH** | ***p* value** |
| --- | --- | --- | --- |
|  | **n = 132** | **n = 1447** |  |
| All 4 variables | 11 | 29 | <0.001 |
| None of the 4 variables | 121 | 1418 |  |

|  | **%** | **95% CI** |
| --- | --- | --- |
| Prevalence | 8.4 | 7.0-9.8 |
| Sensitivity | 8.3 | 4.2-14.4 |
| Specificity | 98 | 97.1-98.7 |
| PPV | 27.5 | 14.6-43.9 |
| NPV | 92.1 | 90.7- 93.4 |
| LR (+) | 4.2 | 2.1-8.1 |
| LR (-) | 0.9 | 0.9-1.0 |
| Concordance | 90.5% | |
| Area under the curve | 0.66 | |
